# Adopting Generative AI in Future Classrooms: A Study of Preservice Teachers’ Intentions and Influencing Factors

**DOI:** 10.3390/bs15081040

**Published:** 2025-07-31

**Authors:** Yang Liu, Qiu Wang, Jing Lei

**Affiliations:** School of Education, Syracuse University, Syracuse, NY 13244, USA; yliu424@syr.edu (Y.L.); wangqiu@syr.edu (Q.W.)

**Keywords:** generative artificial intelligence (GenAI), Technology Acceptance Model (TAM), teacher education, behavioral intention

## Abstract

This study investigated pre-service teachers’ (PTs) intentions to adopt generative AI (GenAI) tools in future classrooms by applying an extended Technology Acceptance Model (TAM). Participants were enrolled in multiple teacher-preparation programs within a single U.S. higher education institution. Through a structured GenAI-integrated activity using Khanmigo, a domain-specific AI platform for K-12 education, PTs explored AI-supported instructional tasks. Post-activity data were analyzed using PLS-SEM. The results showed that perceived usefulness (PU), perceived ease-of-use (PEU), and self-efficacy (SE) significantly predicted behavioral intention (BI) to adopt GenAI, with SE also influencing both PU and PEU. Conversely, personal innovativeness in IT and perceived cyber risk showed insignificant effects on BI or PU. The findings underscored the evolving dynamics of TAM constructs in GenAI contexts and highlighted the need to reconceptualize ease-of-use and risk within AI-mediated environments. Practically, the study emphasized the importance of preparing PTs not only to operate AI tools but also to critically interpret and co-design them. These insights inform both theoretical models and teacher education strategies, supporting the ethical and pedagogically meaningful integration of GenAI in K-12 education. Theoretical and practical implications are discussed.

## 1. Introduction

### 1.1. Urgency and Complexity for Pre-Service Teachers with GenAI

The rapid advancement of generative artificial intelligence (GenAI) technologies such as ChatGPT, Claude, and Gemini has signaled a new era in digital education. These tools, capable of generating coherent text, solving complex problems, and simulating human-like conversation, have become increasingly relevant to educational scenarios (Hou et al., 2025; L. Li et al., 2024). While such advances promise pedagogical innovation, the integration of GenAI in classroom settings introduces novel instructional, ethical, and epistemological challenges (L. Li et al., 2024). These challenges are particularly acute for pre-service teachers (PTs), whose beliefs about teaching and technology are still forming and whose professional practices are highly shaped by purposefully designed instructions and training (Guan et al., 2025; Lei, 2009).

Importantly, the instructional use of domain-specific GenAI tools presents distinct affordances and challenges compared to general-purpose large language models. Unlike ChatGPT or Gemini, which are often framed as open-ended assistants (Menon & Shilpa, 2023), tools such as Khanmigo are designed for educational purposes, which aligns with general K–12 curricula and support instruction-specific tasks. For PTs, navigating these tools requires not only technical familiarity but also pedagogical reasoning to ensure alignment with learning goals, classroom management strategies, and equity considerations.

Several challenges complicate this integration. First, PTs must develop the capacity to critically assess GenAI-generated content for accuracy, appropriateness, and educational value, which is often underemphasized in teacher preparation (Pei et al., 2025). Second, concerns about algorithmic bias, data privacy, and student autonomy require PTs to adopt an ethical lens when deploying GenAI tools (Selwyn, 2022). Third, the novelty and perceived complexity of GenAI systems may lead to apprehension or resistance among PTs, particularly when they lack structured opportunities for guided exploration in authentic instructional contexts (Liu & Lei, 2025). These challenges underscore the importance of embedding domain-specific GenAI tools within pedagogically meaningful teacher education experiences.

### 1.2. Pre-Service Teachers’ AI Adoption Intention

The Technology Acceptance Model (TAM), introduced by Davis (1989), has served as a solid theoretical framework for predicting individuals’ adoption of new technologies. At its core, TAM posits that perceived usefulness (PU) and perceived ease-of-use (PEU) influence an individual’s behavioral intention (BI) to use a given technology. The model has been widely applied across domains including education and has evolved to include additional variables, such as self-efficacy (SE), perceived risk, and enjoyment, to reflect the complex cognitive and emotional evaluations involved in digital tool adoption (Venkatesh & Davis, 2000; Teo, 2012). Notably, TAM offers a structured lens through which to explore how PTs cognitively process the benefits and challenges of such tools, how emotionally engaging they find them, and how these experiences shape their intention to incorporate AI into their future teaching (C. Zhang et al., 2025b).

A growing number of recent international studies have begun to explore PTs’ relationships with AI, but the focus, population, and methodological design vary. Sanusi et al. (2024) applied the theory of planned behavior to examine behavioral factors influencing Nigerian PTs’ AI acceptance, finding basic knowledge and subjective norms to be key predictors of intention to learn AI, though the study did not focus on actual tool use or classroom integration. Runge et al. (2025) applied TAM to study German PTs’ AI acceptance, emphasizing the role of AI-TPACK and teacher training courses. C. Zhang et al. (2023) conducted a large-scale TAM-based multigroup analysis in Germany, revealing gendered patterns in constructs like AI anxiety and perceived enjoyment. A few studies investigated Chinese PTs’ BI towards AI. For instance, Yao and Wang (2024) explored pre-service special education teachers’ intentions to adopt AI tools, highlighting the role of digital literacy and teacher self-efficacy. C. Zhang et al. (2025a) used a latent profile analysis to identify subgroups of AI acceptance among Chinese PTs, emphasizing the impact of digital literacy and compatibility concerns.

While these studies contribute valuable perspectives, there remains a critical gap in understanding how U.S. PTs engage with GenAI across cognitive, affective, and behavioral dimensions, particularly within teacher education programs, where exposure to AI may still be inconsistent or superficial. A recent meta-analysis by McGehee (2024) synthesized 60 studies examining educators’ acceptance of AI technology in education, confirming the widespread use of the TAM and common constructs such as PU, ease-of-use, enjoyment, and risk. However, there are several important distinctions between those studies and the current research. First, nearly all studies reviewed focused on general-purpose large language models (LLMs), such as ChatGPT, rather than domain-specific GenAI tools tailored to K-12 instructional settings. This limits the ecological validity of the findings for classroom-based education. Second, most studies examined in-service teachers, rather than PTs in formal training, and fewer still focused on participants enrolled in technology integration courses. Third, most studies assessed generalized attitudes or perceptions through cross-sectional surveys, without embedding AI use in a specific pedagogical context.

### 1.3. The Current Study

The present study was among the first to investigate how PTs interacted with Khanmigo, a domain-specific GenAI hub explicitly designed to support K-12 teaching and learning. This interaction took place within a structured, pedagogically framed technology integration course, providing a more authentic and purpose-aligned context than studies based solely on hypothetical or informal use. This design enabled a deeper understanding of PTs’ cognitive, affective, and behavioral responses to GenAI, grounded in actual instructional tools rather than abstract perceptions of LLMs. Accordingly, the study contributes a novel and much-needed perspective that addresses significant gaps in both theoretical and applied GenAI research in teacher education.

To explore these dynamics, the study extended the TAM to examine the factors that predicted PTs’ intention to adopt GenAI tools in their future teaching. Originally developed by Davis (1989), TAM posits that PU and PEU are central to forming BI. Building on TAM’s core, the present research incorporated five additional constructs reflecting the affective, cognitive, and dispositional dimensions relevant to AI contexts: SE, perceived enjoyment (PE), perceived cyber risk (PCR), and personal innovativeness in IT (PIIT). Together, these variables provided a multi-dimensional framework for understanding how PTs made sense of GenAI in instructional settings. Based on this framework, the study addresses two research questions:

1. What factors from the Technology Acceptance Model and its extensions predict pre-service teachers’ behavioral intention to use generative AI tools in their future teaching practice?
2. What are the direct and indirect effects of cognitive, affective, and dispositional variables on pre-service teachers’ behavioral intention to adopt generative AI tools in their future instructional practice?

## 2. Theoretical Foundation of Influencing Factors

The emergence of GenAI systems such as ChatGPT, Gemini, and Khanmigo introduces new affordances and epistemological complexities that challenge the traditional assumptions embedded in TAM. These tools are not merely passive instruments but active generators of content, prompting interactions that are adaptive, conversational, and often unpredictable (Mills et al., 2024). To advance TAM in the context of GenAI adoption in teacher education, this study employed an extended TAM framework adapted from Al-Adwan et al. (2023). This included the core TAM constructs (PU, PEU, and BI) and incorporated four additional variables: SE, PE, PCR, and PIIT. These variables were selected based on their relevance to the affective, cognitive, and dispositional dimensions of human and AI interaction, particularly within instructional settings.

**Figure 1:**
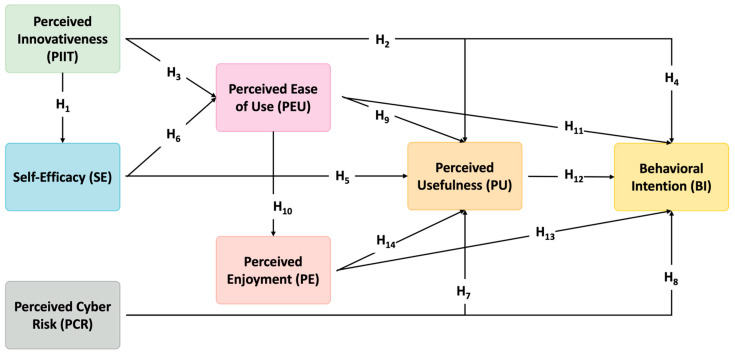
Theoretical hypotheses model.

By situating these constructs within a TAM-based structural model, this study aims to capture the complex interplay between individual dispositions, emotional responses, and technology perceptions that inform GenAI adoption. This multidimensional model also aligns with recent calls to contextualize TAM within AI-mediated educational environments (McGehee, 2024; Venkatesh & Davis, 2000). The following subsections elaborate on each factor’s theoretical relevance and hypothesized effects. Figure 1 shows the theoretical hypotheses model.

### 2.1. Personal Innovativeness in IT (PIIT)

Personal innovativeness in IT (PIIT) refers to an individual’s willingness to try out new information technologies, independently of external influence (Agarwal & Prasad, 1998). It reflects an openness to technological experimentation and change, often marking individuals as early adopters in the diffusion of innovation processes (Rogers, 2003). PIIT is increasingly seen as a critical construct for understanding how teachers and PTs engage with digital tools such as Virtual Reality and, increasingly, AI (Gupta & Bhaskar, 2020, 2023).

PIIT was found to shape a variety of technology-related perceptions and behaviors. Individuals with high PIIT are often more confident in their technological abilities and experience less computer-related anxiety, which in turn positively affects their SE (Bubou & Job, 2022).

PIIT also plays a major role in shaping how users perceive the usability of technology. Innovative users tend to have higher digital fluency and are more inclined to perceive digital tools as easy to use because they possess the skills and mindset necessary to explore them efficiently (Yi et al., 2006). Multiple studies in domains such as mobile learning and virtual environments confirm that PIIT is positively correlated with PEU or effort expectancy (Al-Adwan et al., 2023; Fatima et al., 2017). Thus, PIIT may foster a general openness and readiness to interact with emerging technologies, which could positively influence how PTs perceive the usability of GenAI.

Beyond usability, PIIT also enhances perceptions of usefulness. When individuals are willing to explore unfamiliar systems and possess the adaptability to contextualize technological affordances, they are more likely to recognize how these tools can enhance task performance, teaching efficiency, or student engagement (W. T. Wang & Lin, 2021). GenAI’s utility can manifest in real-time feedback, automated support, or lesson personalization (Walter, 2024); those with high PIIT are more inclined to interpret these features as professionally valuable.

Moreover, PIIT has been consistently identified as a significant predictor of BI to adopt educational technologies, including eMobile-learning platforms and AI-powered chatbots (Chen, 2022; Yao & Wang, 2024). In current teacher education, where instructional support for experimenting with GenAI is often limited (Pei et al., 2025), PIIT may be especially important in enabling PTs to overcome uncertainty and commit to future classroom integration. This is because PIIT may reflect a deeper conceptual engagement with the epistemological and pedagogical implications of AI. Accordingly, we extended the TAM framework by integrating PIIT to examine whether dispositional readiness continues to serve as a distinguishing factor in GenAI environments. Thus, we proposed the following hypotheses:

**H1.**  *PIIT has a positive influence on SE.*

**H2.**  *PIIT has a positive influence on PU.*

**H3.**  *PIIT has a positive influence on PEU.*

**H4.**  *PIIT significantly and positively affects PTs’ BI to use GenAI in teaching practice.*

### 2.2. Self-Efficacy (SE)

Self-efficacy (SE) refers to an individual’s belief in their ability to execute tasks successfully and influence outcomes through their actions (Bandura, 1997). In digital learning environments, SE was shown to significantly shape how learners engage with technology, affecting not only their confidence but also their persistence and resilience in the face of challenges (Scherer et al., 2019).

Higher levels of SE have been consistently associated with more positive perceptions of technology’s usability and usefulness (Venkatesh & Davis, 1996). PTs with strong SE are more confident in their ability to explore and utilize emerging technologies such as GenAI, which may include the ability to craft prompts, interpret AI responses, or evaluate AI-generated content (K. Wang et al., 2024). As such, they are more likely to perceive these tools as easy to use and beneficial for instructional purposes. Recent findings also link SE to BI in AI contexts. For instance, a meta-analysis by McGehee (2024) highlighted SE as one of the most robust and consistent predictors of AI acceptance across educational studies. Although these studies emphasize intention, they indirectly support the broader influence of SE on perceptions of usability and value, which are foundational constructs in TAM. This research considers SE as a factor influencing PTs’ use of GenAI. The following hypotheses are proposed:

**H5.**  *SE has a positive influence on PU.*

**H6.**  *SE has a positive influence on PEU.*

### 2.3. Perceived Cyber Risk (PCR)

Perceived cyber risk (PCR) refers to the anticipated negative consequences, such as data breaches, privacy violations, and misuse of personal information, that users associate with adopting a particular digital technology (S. Li & Gu, 2023). While traditional TAM frameworks do not explicitly account for risk, numerous scholars have emphasized the need to integrate perceived risk as a critical inhibitor of technology adoption, especially in data-sensitive environments like education (C. Zhang et al., 2025a). In the context of GenAI, cyber risk is amplified by the opaque nature of AI systems, the volume of user data collected, uncertainties about how AI stores and processes user input data, and the uncertain outcomes produced by algorithmic reasoning (Selwyn, 2022). When educators fear potential disruptions to classroom management, damage to their professional image, or risks of student data misuse, they are less likely to view AI tools as useful (Hu et al., 2025). Research in adjacent domains, such as the metaverse, has also shown that higher perceived risk leads to lower PU and BI (Al-Adwan et al., 2023).

Therefore, understanding how PTs perceive and respond to cyber risks is imperative, particularly as they navigate an emerging AI-mediated educational landscape without consistent institutional support or clear policy (Diliberti et al., 2024). Therefore, we proposed the following hypotheses:

**H7.**  *PCR significantly and negatively affects PU.*

**H8.**  *PCR significantly and negatively affects PTs’ BI to use Gen AI in teaching practice.*

### 2.4. Perceived Ease-of-Use (PEU) and Perceived Usefulness (PU)

Perceived ease-of-use (PEU) and perceived usefulness (PU) are the core factors in various TAM versions (Davis, 1989; Venkatesh & Davis, 2000). According to Davis (1989), PEU is defined as the degree to which an individual believes that using a particular technology will be free of effort. PU is defined as the degree to which an individual believes that using a specific technology will enhance their job performance.

Research consistently identifies PEU as a significant antecedent of PU and BI in various educational technology contexts, suggesting that technologies which reduce users’ cognitive friction are more likely to be seen as valuable and to be adopted (Davis et al., 1992; Scherer et al., 2019). Moreover, PEU is also linked to PE, as users are more likely to enjoy systems that are convenient and require less effort to operate (Tam et al., 2020).

PU is frequently reported as one of the strongest predictors of BI in TAM studies, influencing both willingness to try and the actual integration of the technology (Venkatesh & Davis, 2000; Scherer et al., 2019). In education, PU often reflects a teacher’s judgment about whether a technology improves the effectiveness of teaching, student learning outcomes, or pedagogical efficiency (Gao et al., 2025). PTs who perceives GenAI as beneficial are more inclined to integrate it into their instructional repertoire. Thus, we proposed the following hypotheses:

**H9.**  *PEU significantly and positively affects PU.*

**H10.**  *PEU significantly and positively affects PE.*

**H11.**  *PEU significantly and positively affects PTs’ BI to use Gen AI in teaching practice.*

**H12.**  *PU significantly and positively affects PTs’ BI to use Gen AI in teaching practice.*

### 2.5. Perceived Enjoyment (PE)

Perceived enjoyment (PE) refers to the extent to which using a technology is perceived to be enjoyable in its own right, aside from any external rewards or outcomes (Abdullah & Ward, 2016; Venkatesh, 2000). Rooted in intrinsic motivation, enjoyment, as a hedonic factor, often shapes learners’ emotional responses (e.g., engagement and satisfaction) to learning platforms, making learning more engaging, playful, and meaningful (Teo & Noyes, 2011).

Research indicates that when users experience enjoyment while using technology, they are more likely to form favorable perceptions about its value and usefulness (X. Zhang et al., 2021). Prior studies also revealed that PE is positively associated with PU (Mun & Hwang, 2003), especially in environments where learners feel emotionally supported and cognitively stimulated. Furthermore, PE has consistently been identified as a strong predictor of BI. Students and educators who find learning systems enjoyable are more motivated to continue using them, which enhances both adoption and sustained engagement (Chocarro et al., 2021). Given the dynamic and dialogic nature of AI interfaces, PTs who find interacting with GenAI tools enjoyable may perceive them as more beneficial to their instructional practice, which in turn strengthens their intention to incorporate them into future classrooms. The proposed hypotheses are as follows:

**H13.**  *PE significantly and positively affects PTs’ BI to use Gen AI in teaching practice.*

**H14.**  *PE significantly and positively affects PTs’ PU.*

## 3. Methodology

### 3.1. Participants

In this study, we recruited 56 PTs from a large private institute from Northeast US who had enrolled in two technology integration courses during the 2024–2025 academic year. Table 1 illustrates the demographic information of participants. Participants consisted of 70% White females, 18% White males, and smaller representations of Asian, Black, and Latinx students. The majority (n = 35) were from an inclusive elementary and special education program, while the remaining participants were from inclusive adolescent education, mathematics education, physical education, and other education majors. A pre-course survey indicated a similar digital literacy level among participants and no prior formal training in generative AI. While most had limited experience using AI for tasks such as text refinement and idea generation, none had previously engaged with the AI-integrated activity designed for this study. Specifically, 98% of participants reported prior experience using AI technologies, with 90% having used ChatGPT and 5.4% being familiar with AI functionalities in tools like Canva or Grammarly. Nearly all participants (98%) reported owning more than one app or platform with embedded AI functionalities, such as Instagram.

### 3.2. Research Design

A Generative AI unit was delivered by the same instructor across both courses. Following an introductory lecture on the role of generative AI in education, participants engaged in a 1.5 h, hands-on, AI-integrated activity. Students completed the pre-survey before the class. After the AI-integrated activity, they completed the post-survey to reflect on their experiences and perceptions.

#### AI-Integrated Activity

The AI-integrated activity was designed for PTs to explore and experiment with Khanmigo tools, a hub of AI-powered instructional resources available on Khan Academy and powered by ChatGPT-4. Khanmigo offers over 25 AI-powered activities that assist teachers in five key aspects of instructional practice: planning, creating, differentiating, supporting, and learning. Each PT freely selected one tool from each of the five categories to experiment with. They interacted with the AI-powered tools by providing specific prompts relevant to their instructional needs, such as a particular teaching scenario or a pedagogical challenge. The participants then documented their input, describing their instructional context and requirements, and recorded the AI-generated outputs provided by the tools.

### 3.3. Measurements

The pre-survey collected background information on participating PTs, including their academic major, prior experience with AI tools, and any previous AI-related training. The post-survey employed an extended TAM instrument incorporating seven constructs—PU, PEU, SE, PE, PCR, PIIT, and BI—adapted from Al-Adwan et al. (2023). Minor modifications were made to the original scale items to ensure contextual relevance to the present study. The final instrument comprised 17 items measured on a 5-point Likert scale, ranging from 1 (strongly disagree) to 5 (strongly agree). A pilot study was conducted to evaluate the internal consistency of each construct. The results indicated that all constructs met the recommended threshold for reliability, with Cronbach’s alpha coefficients exceeding 0.70 (Hair et al., 2022). Specifically, Cronbach’s alpha coefficients for PU, PEU, SE, PE, PCR, PIIT, and BI were 0.74, 0.77, 0.71, 0.78, 0.71, 0.75, and 0.79, respectively. To further establish the validity of the contents, a panel of four scholars with expertise in educational technology reviewed the instrument. A detailed list of survey items is provided in Appendix A.

### 3.4. Data Analysis

The technique chosen for data analysis in this study was Partial Least Squares Structural Equation Modeling (PLS-SEM). PLS-SEM is a flexible method that can be utilized in various settings, and it has less stringent requirements for sample size and distribution compared to other modeling techniques (Hair et al., 2022). We analyzed the data using SmartPLS 4.1.1.2 software (Ringle et al., 2022). Following the recommended procedures, we conducted the data analysis in a stepwise manner (Anderson & Gerbing, 1988). In the first step, we evaluated the measurement model by assessing internal consistency, as well as convergent and discriminant validity. After confirming that the results from this step were acceptable, we proceeded to step two, where we applied the structural model to test our hypothesis.

## 4. Results

### 4.1. Preliminary Data Check

Prior to conducting the structural analysis, we assessed the possibility of multicollinearity by examining variance inflation factor (VIF) values for all individual indicators. Following Hair et al. (2022), a VIF threshold of 3.0 was adopted to signal potential multicollinearity issues. All items reported VIF values well below this threshold, ranging from 1.156 (PU3) to 1.968 (PE1 and PE2). These results demonstrated that indicator-level multicollinearity is not a concern, and each measurement item contributes uniquely to its corresponding latent construct. To assess the presence of common method bias (CMB), we employed the Full Collinearity VIF approach proposed by Kock (2017). This method involves evaluating the VIF for all latent variables in the structural (inner) model, with a threshold of 3.3 indicating the presence of potential method bias. As shown in the VIF results (see Table 2), all VIF values were well below the recommended cut-off. These results confirmed that CMB was absent in this study.

### 4.2. Measurement Model

Before examining the proposed hypotheses, the reliability and validity of the measurement items (indicators) and scales (constructs) were evaluated, following the guidelines by Hair et al. (2022). First, indicator reliability was assessed through the outer loadings. A factor loading of 0.708 or higher is generally considered acceptable. As shown in Table 3, all items demonstrate loadings above or near this threshold. The lowest loading was observed for PU3 (0.693), which is marginally below the cutoff. However, this value is still within the acceptable range (≥0.60) when the construct’s composite reliability and Average Variance Extracted (AVE) are satisfactory (Hair et al., 2022). Therefore, all items were retained. Next, internal consistency was examined using Cronbach’s Alpha (α) and Composite Reliability (CR). All constructs met the minimum recommended value of 0.70 for both α and CR, with none exceeding the upper threshold of 0.95, indicating no redundancy among items. This demonstrates strong internal consistency across all constructs.

Next, convergent validity was assessed by examining the AVE, where a value of 0.50 or above is considered acceptable. The AVE values for all constructs ranged from 0.573 to 0.850, exceeding the 0.50 benchmark and confirming the presence of convergent validity. The cross-loading analysis showed that each item loaded more strongly on its intended construct than on any other constructs, further supporting the discriminant and convergent validity of the measurement model.

Discriminant validity was assessed using both the Fornell–Larcker criterion and the Heterotrait–Monotrait Ratio (HTMT) approach. According to Fornell and Larcker (1981), the square root of the average variance extracted (√AVE) for each construct should be greater than its correlations with any other construct. As shown in Table 4, this condition was met for all constructs. For example, the √AVE of PU is 0.757, which exceeds its correlation with all other constructs, including BI at 0.667 and PE at 0.599. These findings confirm that the Fornell–Larcker criterion is satisfied, indicating sufficient discriminant validity under this approach.

To complement this assessment, the HTMT values were examined. HTMT values below 0.85 are generally considered acceptable (Henseler et al., 2015). As reported in the upper triangle of Table 3, most HTMT values fell below this threshold, supporting discriminant validity. However, one was observed between BI and PU with an HTMT value of 0.896, which exceeds the 0.85 threshold; this exception may indicate potential overlap between these constructs. Although the conventional HTMT threshold is 0.85, several scholars proposed a more relaxed threshold of 0.90, especially in exploratory research or when the constructs in question are theoretically or conceptually related (Hair et al., 2022). In the present study, the relatively high HTMT value between BI and PU can be considered acceptable under the 0.90 threshold. Moreover, it is theoretically justified, as PU is a direct antecedent of BI in well-established models such as the TAM, which may naturally lead to a stronger empirical association.

### 4.3. Structural Model

**Figure 2:**
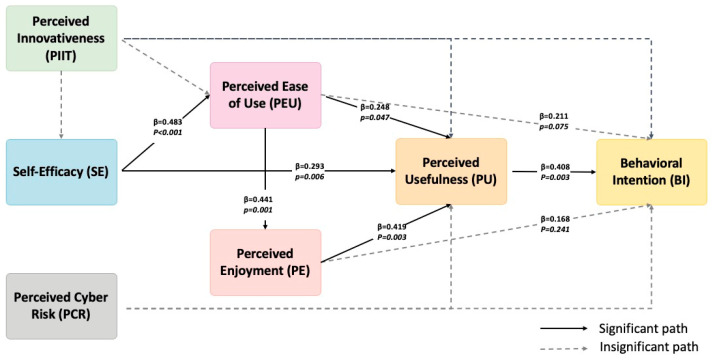
Path model with direct effect.

To test the proposed hypotheses, a bootstrapping procedure with 5000 resamples was conducted. Table 5 and Figure 2 summarize the standardized path coefficients, confidence intervals, and significance levels for all hypothesized relationships.

Among the 14 hypothesized paths, 7 were statistically supported at the 0.05 significance level. Specifically, SE showed significant positive effects on both PEU (β = 0.483, *p* < 0.001) and PU (β = 0.293, *p* < 0.01), supporting H5 and H6. Similarly, PEU had significant effects on PE (β = 0.441, *p* < 0.005) and PU (β = 0.248, *p* < 0.05), confirming H9 and H10. These results implied that PEU has an important role in boosting PU and PE. Furthermore, PU significantly influenced BI (β = 0.408, *p* < 0.005), supporting H12. Lastly, PE had a significant positive influence on PU (β = 0.419, *p* < 0.005), supporting H14.

On the other hand, several paths were found to be non-significant. Notably, PIIT had no significant direct effects on SE, PU, PEU, or BI (H1–H4, all *p* > 0.05). Similarly, PCR did not significantly influence either PU or BI (H7 and H8, all *p* > 0.05). The path from PEU to BI (H11) also failed to reach significance (*p* > 0.05), suggesting a limited direct effect on BI in this context. Additionally, PE did not significantly influence BI (H13, *p* > 0.05), even though it strongly affected PU.

In terms of the explanatory power of the model, the R^2^ values provide insight into how well the predictor constructs explain the variance in the endogenous variables. The model explains 54.8% of the variance in BI, 54.0% in PU, 23.2% in PEU, and 19.5% in PE. These values suggest that the model has moderate to substantial predictive power (Hair et al., 2022), particularly in explaining PTs’ intention and PU, which are central constructs in the adoption process of generative AI technologies.

Overall, these results provided partial support for the hypothesized model, highlighting the key roles of PU, PEU and SE in shaping PTs’ BI to use Gen AI, while indicating limited influence from PIIT and PCR within this context. As proposed, PCR serves as a key obstacle of students’ BI regarding Gen AI adoption and regarding PU. However, it displayed insignificant effects on BI and PU, which was contradictory to the original proposal.

To further assess the relative contribution of each exogenous variable to the explanatory power of the endogenous constructs, effect sizes (*f^2^*) were calculated. According to Cohen’s (1988) guidelines, *f*^2^ values of 0.02, 0.15, and 0.35 are considered small, medium, and large, respectively.

As shown in Table 6, several predictors demonstrated meaningful effect sizes. Notably, PU had a medium effect on BI (*f*^2^ = 0.193), suggesting its substantial role in influencing users’ intention to adopt generative AI. Similarly, PEU exhibited a medium-to-large effect on PE (*f*^2^ = 0.242), and SE had a large effect on PEU (*f*^2^ = 0.296), highlighting their significant predictive power. On the other hand, some predictors showed only small or negligible effects. For instance, PCR and PE had small effects on BI (*f*^2^ = 0.045 and 0.035, respectively), while PIIT’s effect on all endogenous variables remained minimal (e.g., *f*^2^ = 0.032 on BI, 0.008 on PU, and 0.026 on SE). These results indicate that although PIIT was included in the model, its direct influence on key constructs is limited in this context.

Overall, the *f*^2^ analysis supports the key roles of PU, PEU, and SE as influential predictors, whereas other constructs, such as PIIT and PCR, contribute comparatively less to the model’s explanatory power.

To ensure sufficient statistical sensitivity for each hypothesis in the proposed model, a Monte Carlo simulation-based power analysis was conducted. This method is particularly appropriate for SEM, especially when working with latent variables, bootstrapped estimates, and relatively small samples. As recommended by Y. A. Wang and Rhemtulla (2021), simulation-based power estimation enables researchers to assess the likelihood of detecting true effects in complex model structures under empirically realistic conditions. Similarly, Hair et al. (2022) emphasized the relevance of Monte Carlo simulations in PLS-SEM for evaluating power and effect robustness, particularly when sample sizes are modest.

Based on 1000 simulations with a sample size of n = 56, the estimated statistical power for each structural path was derived using standardized beta coefficients from the final PLS-SEM model. The results showed that four paths achieved sufficient power levels (≥0.80): SE → PEU (β = 0.483, power = 0.922), PEU → PE (β = 0.441, power = 0.890), PE → PU (β = 0.419, power = 0.832), and PU → BI (β = 0.408, power = 0.835). These findings indicated a high probability of detecting meaningful effects in these hypothesized relationships. Although both the SE → PU and PEU → PU paths reached statistical significance in the structural model, the power analysis revealed relatively low sensitivity for detecting these direct effects (power = 0.568 and 0.430, respectively). In fact, these results suggested that a more powerful mediation pathway, SE → PEU → PE → PU → BI, demonstrated higher power across each component path (ranging from 0.832 to 0.922) and yielded statistically significant indirect effects. A more powerful affective–cognitive mediation structure was revealed. This pattern indicates that the influence of SE and PEU on BI may be more robustly transmitted through a cascading mediation mechanism, wherein PE plays a central role in shaping PU and, ultimately, BI.

### 4.4. Indirect Effect Assessment

To examine the mediating mechanisms underlying PTs’ BI to use generative AI in their future teaching practice, both total and specific indirect effects were analyzed to reveal the influence of mediating variables embedded in the TAM-based structural model. The significance of the indirect effects of the research model’s constructs is shown in Table 7.

The results revealed several statistically significant total indirect effects. SE demonstrated a strong and significant total indirect effect on BI (β = 0.342, *p* < 0.001), indicating that SE influenced BI entirely through mediators such as PU (PU), PEU, and PE. Additionally, PEU had a significant total indirect effect on BI (β = 0.251, *p* < 0.005), and on PU (β = 0.185, *p* < 0.05), while SE also indirectly influenced both PU (β = 0.209, *p* < 0.05) and PE (β = 0.213, *p* < 0.05) via PEU.

In terms of specific indirect effects, three indirect paths reached significance. SE had a significant indirect effect on BI through PU (SE → PU → BI; β = 0.119, *p* < 0.05), suggesting that participants with higher confidence in their ability to engage with AI-based tools were more likely to perceive AI as useful, which in turn promoted their intention to adopt it in teaching. Moreover, SE positively influenced PE via PEU (SE → PEU → PE; β = 0.213, *p* < 0.05), implying that ease of use perceptions enhanced the enjoyment experienced during AI-integrated learning. We also found that PEU significantly influenced PU through the PEU → PU pathway (β = 0.185, *p* < 0.05), further reinforcing the TAM assumption that ease of use contributes to usefulness perception.

Although some specific mediation pathways (e.g., PIIT → PEU → BI or SE → PEU → BI) did not reach statistical significance, their inclusion remains theoretically informative and provides a more nuanced understanding of how PTs process new technologies like GenAI when applied to future instructional settings.

These findings affirmed the critical role of both cognitive (i.e., PU and PEU) and affective (i.e., PE) evaluations in mediating the relationship between internal traits, particularly SE and BI to integrate GenAI into K12 teaching. These results aligned with the core assumptions of the TAM, which posited that these perceptions of utility and usability function as key mechanisms shaping technology adoption.

### 4.5. Explore the PTs’ BI Using Classic TAM Without Considering the PCR and PIIT

**Figure 3:**
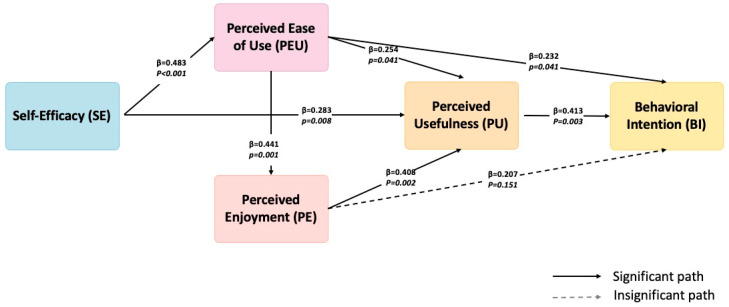
Classic TAM direct and indirect effects.

To further isolate the explanatory power of the original TAM, we conducted a secondary analysis excluding the constructs of PCR and PIIT. As illustrated in Figure 3, this classic TAM, comprising SE, PEU, PU, PE, and BI, resulted in improved model clarity and increased path significance, particularly for the link between PEU and BI (β = 0.232, *p* < 0.05), which was previously non-significant. It appears that, in this study, classic TAM constructs, particularly SE, PEU, PU, and PE, sufficiently account for the motivational and cognitive–affective processes driving PTs’ intention to adopt generative AI in pedagogical settings. This supports prior research showing that TAM’s core tenets remain robust in digital-native teacher populations (Teo, 2012; Davis, 1989), particularly when the technology in question is becoming increasingly integrated into mainstream discourse and educational experimentation.

## 5. Discussion and Implications

This study contributes to the growing body of research on generative AI in education by advancing both theoretical understanding and practical application. Theoretically, the findings extend the TAM by illuminating the interdependent roles of PU, PEU, and SE in shaping BI. In particular, the affective pathway from PE to PU highlights the importance of emotional engagement in evaluating GenAI tools, moving beyond traditional, utility-driven interpretations of technology adoption. Additionally, the non-significant effects of PIIT and PCR reveal important boundary conditions for TAM constructs in GenAI contexts. These results suggest that high baseline digital fluency and educational scaffolding may diminish the predictive power of traditional individual-difference variables. At the same time, they uncover the complex and evolving nature of GenAI adoption, shaped by factors such as emotional engagement, the contextual framing of AI use, the characteristics of the AI platform, and the design of teacher education programs. These factors collectively influence technology acceptance in ways that extend beyond individual psychological traits.

Practically, the study highlights the importance of moving beyond general AI literacy to address specific areas that are essential for effective GenAI integration. These areas include instructional strategies to enhance perceived ease of use through scaffolded prompt design, the cultivation of emotional engagement to increase perceived enjoyment, the development of epistemic and pedagogical understanding to address the limitations of personal innovativeness in IT, and the promotion of algorithmic transparency to foster risk awareness. Incorporating these elements into teacher education curricula can help PTs build the necessary competence, critical thinking, and ethical judgment to engage with GenAI tools in responsible and pedagogically meaningful ways.

### 5.1. Increasing Interdependence of Core TAM Constructs

A key contribution of this study lies in uncovering the dynamic interdependence among core TAM constructs within the context of generative AI adoption. Rather than merely reaffirming the relevance of PU, PEU, and SE, the findings highlight how these factors interact in meaningful and layered ways. Specifically, SE significantly enhanced PEU, which in turn influenced both PE and PU, ultimately shaping BI. This cascading pattern suggests that PTs’ technology adoption decisions are not based on isolated evaluations but emerge from an interconnected web of cognitive and affective appraisals.

This interdependence aligns with recent shifts in the educational technology literature that conceptualize technology acceptance as a systems process, where constructs such as ease of use and usefulness are co-constructed through personal confidence and emotional experience (Shen et al., 2022; Venkatesh et al., 2012). In GenAI-enhanced learning environments, these relationships appear to be intensified. For instance, PEU is not simply a matter of technical ease, but a reflection of one’s SE with AI-mediated dialog, which in turn enhances both enjoyment and perceived instructional utility.

Moreover, this finding positions PU, PEU, and SE not just as predictors of BI, but as evolving literacies that are foundational to future teaching. As AI becomes increasingly embedded in lesson planning, formative assessment, and pedagogical design, these constructs represent core professional capacities for ethical and productive navigation in AI-supported classrooms (U.S. Department of Education, 2023, 2024). By foregrounding the interdependence of these constructs, this study moves beyond conventional TAM validation to offer a more nuanced, process-oriented understanding of GenAI adoption in teacher education.

### 5.2. The Central Role of PU Shaped by Affective Experience

PU was found to be the most influential direct predictor of BI. This finding aligned with the foundational propositions of TAM, where perceived instrumental value is central to the adoption decision (Venkatesh & Davis, 2000). In this study, the generative capacities of AI tools like Khanmigo were viewed by many PTs as pedagogically relevant, which reinforced the PU construct.

Notably, the structural path revealed a strong link between PE and PU, suggesting that in GenAI environments, cognitive judgments about usefulness are often shaped by affective responses during tool interaction. Unlike traditional digital tools that follow deterministic scripts, GenAI systems such as Khanmigo engage users through adaptive, conversational, and sometimes unpredictable interactions. These interactions evoke curiosity, playfulness, and a sense of discovery, which can elevate the perceived instructional value of the tool. In this way, enjoyment becomes more than an emotional byproduct; it functions as a cognitive amplifier that informs judgments of utility (F. Wu et al., 2025).

Through the power analysis, our findings revealed that the indirect pathways, particularly, SE → PEU → PE → PU → BI, were not only statistically significant but also demonstrated a consistently strong statistical power. This pattern points to a meaningful cascading mediation process, in which SE enhances PEU, which in turn fosters PE. This affective engagement strengthens PU, ultimately shaping BI. Rather than acting through isolated direct paths, SE and PEU appear to exert their influence through an interconnected chain of cognitive and affective appraisals. This layered structure highlights the importance of PE as a bridge between PEU and PU, especially in emotionally engaging contexts like GenAI-supported learning. This affective shaping of PU highlights an important affect-to-cognition transmission in GenAI adoption. This suggests that, in GenAI-enhanced learning environments, emotional engagement is not peripheral but central to how PTs evaluate technological value. By foregrounding the influence of PE on PU, this study adds to a growing body of evidence that affective experiences play a formative role in shaping educators’ cognitive appraisals and eventual adoption decisions (McGehee, 2024), particularly for tools that rely on human–AI interaction.

### 5.3. Self-Efficacy as a Foundational Enabler

SE demonstrated significant effects on both PEU and PU and had an indirect influence on BI through these constructs. This finding supports Bandura’s (1997) view that confidence in one’s ability to perform a task increases one’s likelihood of perceiving it as manageable and valuable. In this case, PTs who felt more capable of engaging with AI tools were more inclined to recognize their instructional utility. These results are consistent with prior research showing that efficacy beliefs are central to teachers’ willingness to adopt emerging technologies (Scherer et al., 2019).

It is important to emphasize that SE in the context of GenAI encompasses more than comfort with digital tools. Rather, it involves the perceived ability to formulate effective prompts, interpret AI-generated outputs, and adapt these outputs to instructional needs (Mills et al., 2024). We believe that SE is an enabling condition for cognitive and affective appraisals alike, supporting both ease and value perceptions.

### 5.4. Redefining PEU in a Prompt-Centric Environment

While PEU did not directly predict BI at a statistically significant level, it significantly influenced both PU and PE. Our findings echoed with Tiwari et al. (2024)’s finding that PEU no longer worked as determinant in AI adoption. However, this partial result reflected a shifting understanding of ease within AI-empowered education. Traditional interpretations of PEU often focused on interface simplicity or technical fluency. In contrast, ease of use with GenAI increasingly relates to one’s ability to effectively interact through prompts and critical interpretation. This evidence suggests that teacher preparation programs should consider expanding their definition of digital literacy to include pedagogical AI prompt design, response evaluation, and an understanding of the probabilistic nature of AI outputs (Kohnke et al., 2025). One practical implication is to improve PEU through structured training in prompt design. As GenAI tools rely heavily on user-inputted prompts to generate meaningful content, PTs must learn how to craft effective, pedagogically aligned prompts that yield desired instructional outputs. Embedding low-stakes exercises on prompt iteration and critique into coursework can increase both technical fluency and pedagogical confidence. Moreover, connecting instructional strategies with the constructs identified in this study to design future interventions may be beneficial, such as paring PEU enhancement activities with reflections or discussion on PE and SE to strengthen both affective engagement and user agency.

### 5.5. Rethinking the Role of Personal Innovativeness in IT (PIIT)

Contrary to prior studies that identify PIIT as a critical antecedent of technology acceptance (Agarwal & Prasad, 1998; W. Wu & Yu, 2022), this study found no significant relationship between PIIT and SE, PU, PEU, or BI. The insignificant relationships may reflect the characteristics of our sample, as participants were already enrolled in a structured technology integration course at a private institute. This relatively homogeneous group may exhibit a ceiling effect in self-reported innovativeness, reducing the discriminant utility of the PIIT construct. Such findings echo prior research indicating that the influence of personal innovativeness may diminish when digital tools are embedded within guided, institutionally scaffolded contexts (Ramnarain et al., 2024).

Recent work also suggests that in digitally saturated environments, PIIT may no longer meaningfully differentiate early adopters (Milutinović, 2022). In our study, 98% of participants reported prior experience using AI technologies. This high baseline of digital fluency suggests that PIIT may function less as a measure of innovativeness and more as an indicator of digital normativity. As Milutinović (2022) argues, high digital literacy among Gen Z populations can obscure individual differences in tech adoption behaviors, flattening PIIT’s predictive value.

Additionally, the accelerated societal diffusion of AI has left little time for deliberation about its appropriate use in K-12 education (U.S. Department of Education, 2023). Many educators are thrust into AI-mediated environments without opportunities to meaningfully explore pedagogical implications, let alone shape the trajectory of integration (Woodruff et al., 2023). However, the nature of GenAI requires a more complex set of cognitive and epistemic skills than traditional technologies. As machine learning models evolve, educators may need to engage not only with interface-level features but also with an understanding of how algorithms operate, make decisions, and adapt to user data. Without opportunities for epistemic engagement with AI, even tech-savvy users may not exhibit strong innovativeness toward its pedagogical applications (Zhai, 2024). Thus, PIIT may be insufficient as a standalone predictor of GenAI adoption intention unless paired with educational interventions that foster deeper conceptual engagement.

### 5.6. When Risk Fails to Matter: The Role of Perceived Cyber Risk (PCR)

Our study found that PCR had no significant influence on either PU or BI to adopt GenAI in instructional practice. This finding runs counter to a well-established body of literature indicating that data privacy concerns and cyber vulnerabilities serve as psychological and behavioral barriers to technology adoption (Al-Emran et al., 2024). The null effect of PCR may be attributed to the instructional environment in which Khanmigo was introduced. Unlike open-ended GenAI platforms, Khanmigo was targeted within an education environment with clear pedagogical framing. As such, participants may have perceived minimal threats to privacy or security, weakening the salience of cyber risk in shaping behavioral intentions. However, given AI’s inherent reliance on large-scale data capture and opaque algorithmic processes, the absence of risk perception is not only unexpected but potentially problematic.

The results may signal a broader perceptual shift among PTs. Extensive prior exposure to AI-powered applications (e.g., Instagram) may have desensitized participants to cyber risks. While 98% had used AI before and 90% had experience with ChatGPT, only 5.4% had previously engaged with AI in formal educational settings. This disconnect suggests that while AI is omnipresent in their daily lives, its risks are not critically processed unless explicitly surfaced through pedagogical framing. A similar trend was found in Ramnarain et al. (2024)’s study, where PTs’ concern about GenAI had no effect on their BI to adopt AI for inquiry-based learning. As such, PCR may not register as a salient factor when evaluating GenAI tools designed by education entities like Khan Academy.

Such findings align with recent studies indicating that GenAI’s black-box nature is often invisible to users in highly scaffolded environments (Selwyn, 2022; Mills et al., 2024). This perceptual gap becomes particularly problematic in education, where epistemic integrity, equity, and learner autonomy are core values. The absence of risk perception may not reflect the absence of risk, but a lack of critical literacy around AI systems.

More recently, U.S. Department of Education (2024) guidelines and UNESCO’s AI Competency Framework for teachers (Miao & Cukurova, 2024) both emphasize the need to prepare teachers to understand, critique, and ethically implement AI. Miao and Holmes (2021) similarly stress that AI literacy must encompass not just usage but also awareness of algorithmic bias, data security, and system limitations.

Given these concerns, the null effect of PCR in this study may indicate a perceptual blind spot rather than a true absence of risk. The lack of critical discourse around AI in formal education, as only 5.4% of participants had previously discussed AI in academic courses, suggests there is a vacuum in which affective and cognitive assessments of AI risk remain underdeveloped (Pei et al., 2025). This mirrors the earlier observation regarding PIIT. In both cases, foundational psychological constructs fail to predict intention not due to irrelevance, but because the structural and pedagogical infrastructure required for these constructs to manifest meaningfully is lacking.

To move forward responsibly, teacher education programs should embed critical AI literacy, not just in terms of usage, but in understanding algorithmic logic, data flow, ethical design, and policy implications (L. Li et al., 2024). Practically, teacher preparation programs can integrate critical reflection activities or case-based discussions into the existing curricula that examine data ethics, algorithmic bias, and the opaque nature of GenAI tools. These activities would help PTs engage with AI as a dynamic and integral part of educational practice. As a result, programs can cultivate not just GenAI adopters, but critically aware educators that are equipped to shape the ethical use of AI in schools.

## 6. Limitations and Future Research

While this study offers timely insights into PTs’ perceptions and intentions regarding generative AI integration, several limitations warrant careful consideration. First, although the sample reflects the demographic and institutional realities of many teacher education programs in the United States today, it was drawn from a single private university and was predominantly composed of White female participants. This demographic concentration may limit the transferability of findings to broader PT populations, particularly those from more diverse or economically marginalized backgrounds. Considering the lack of variation in PIIT scores among participants, future research should therefore examine the contextual boundaries of PIIT’s explanatory power and consider alternative constructs better suited to capturing variation in more digitally heterogeneous groups, such as epistemic curiosity (Puerta-Sierra & Puente-Díaz, 2024), AI self-agency, or pedagogical trust (Celik et al., 2025). These constructs may offer deeper insight into how teachers navigate GenAI adoption in diverse educational contexts. Nevertheless, this distribution also mirrors the composition of many teacher preparation programs nationwide, and thus sheds light on how GenAI is currently being encountered within mainstream U.S. teacher education.

The relatively small sample size of 56 PTs presents inherent constraints on statistical power and generalizability. Although PLS-SEM is well-suited for small samples (Hair et al., 2022), concerns about detecting medium-to-small effects remain. To address this, we conducted a formal Monte Carlo simulation-based post hoc power analysis (Y. A. Wang & Rhemtulla, 2021). The results showed that most structural paths (e.g., SE → PEU, PEU → PE, PE → PU, PU → BI) achieved adequate power (≥0.80), supporting the model’s sensitivity to hypothesized effects. While the SE → PU and PEU → PU paths reached statistical significance in our model, the accompanying power estimates were relatively modest. These results suggest that although these direct effects are empirically supported, their stability may vary depending on sample characteristics or study design. While these effects are theoretically meaningful and statistically significant in this study and even reveal a more powerful affect-to-cognition transmission and complex mediation pathway, they should nonetheless be interpreted with caution and validated in future research with larger samples. Moreover, the study’s homogeneous composition limited the ability to examine demographic moderating effects. Future work should prioritize larger and more diverse and culturally varied samples across public and private institutions, enabling subgroup analyses and greater external validity. Mixed-method designs, including reflective interviews or longitudinal classroom data, may also provide richer, contextualized insights into how PTs’ attitudes toward GenAI evolve over time and across implementation contexts.

Additionally, the instructional exposure to GenAI in this study occurred within a single activity session. While valuable as a design-based introduction, it does not fully reflect the complex and sustained learning required to build AI fluency for classroom practice. Future research would benefit from extended interventions that trace PTs’ evolving perspectives, experimentation patterns, and the real-world application of AI tools over time. Such work is essential for informing both the theoretical models we use to explain technology adoption and the practical frameworks we use to prepare teachers for an AI-enriched educational landscape.

## 7. Conclusions

This study contributes to a deeper understanding of PTs’ adoption of GenAI tools in educational settings by applying and extending the TAM. Through a detailed structural analysis, the findings reaffirm the central roles of PU, PEU, and SE in shaping BI, while also revealing the limited predictive power of PIIT and PCR within the GenAI context. These results have important theoretical implications. They demonstrate that while TAM’s core constructs remain valid, their operational dynamics are evolving in response to the unique epistemic and interactive features of AI-powered systems. Moreover, the findings emphasize the need to reconceptualize constructs such as ease of use and perceived risk in ways that better reflect AI’s black-box nature, its learning-based architecture, and the sociotechnical environments in which it is deployed. Practically, the study highlights the urgency of equipping teacher candidates not only with the technical skills to use AI tools, but also with the critical literacies necessary to interrogate, adapt, and co-design these systems in educationally meaningful ways. Together, these insights suggest a recalibration of both theoretical models and teacher preparation strategies is needed to ensure the ethical and effective integration of GenAI in K-12 classrooms.

## Figures and Tables

**Table 1:** Demographic characteristics of participants (n = 56).

Characteristic	Category	Frequency (n)	Percentage (%)
Age Range	19–24 years old	-	-
Gender	Female	45	80.4%
	Male	12	21.4%
Race	White	49	87.5%
	Asian	4	7.1%
	Black or Latinx	3	5.4%
Program	Inclusive elementary and special education	35	62.5%
	Inclusive adolescent education	10	17.9%
	Math education	5	8.9%
	Physical education	3	5.4%
	General education (selected subject, e.g., music)	3	5.4%
Prior Teaching	With prior field experience	43	76.8%
	Without prior field experience	13	23.2%
Prior Use of AI Technologies	Yes	55	98.2%
	No	1	1.8%
AI Tools Used	ChatGPT	50	89.3%
	Canva/Grammarly	4	5.4%
	Other	2	3.6%

**Table 2:** Multi-collinearity assessment.

Construct	BI	PEU	PU
PCR	1.292		1.294
PE	1.796		1.435
PEU	1.539		1.537
PIIT	1.067	1.026	1.086
PU	1.907		
SE		1.026	1.337

**Table 3:** Internal and convergent validity assessment.

Construct	Item	Loading	Cronbach’s Alpha (α)	rho_A	Composite Reliability (CR)	AVE
PU	PU1	0.807	0.727	0.736	0.800	0.573
	PU2	0.766				
	PU3	0.693				
PEU	PEU1	0.834	0.749	0.749	0.804	0.582
	PEU2	0.835				
	PEU3	0.796				
SE	SE1	0.871	0.699	0.699	0.832	0.713
	SE2	0.816				
PE	PE1	0.934	0.824	0.837	0.919	0.850
	PE2	0.910				
PCR	PCR1	0.865	0.715	0.719	0.838	0.722
	PCR2	0.833				
PIIT	PIIT1	0.893	0.755	0.755	0.891	0.803
	PIIT2	0.899				
BI	BI1	0.830	0.793	0.797	0.878	0.706
	BI2	0.86				
	BI3	0.831				

**Table 4:** Discriminant Validity.

Construct	BI	PCR	PE	PEU	PIIT	PU	SE
BI	**0.840**	0.551	0.673	0.710	0.050	0.896	0.295
PCR	0.384	**0.850**	0.603	0.513	0.429	0.525	0.266
PE	0.555	0.432	**0.922**	0.571	0.211	0.815	0.392
PEU	0.557	0.291	0.441	**0.763**	0.120	0.840	0.761
PIIT	−0.015	0.234	0.169	0.069	**0.896**	0.228	0.245
PU	0.667	0.285	0.599	0.570	0.070	**0.757**	0.713
SE	0.210	0.154	0.275	0.482	0.160	0.517	**0.844**

Note. Fornell–Larcker criterion (below the main diagonal). Heterotrait–Monotrait Ratio (HTMT) (above the main diagonal). Main diagonal: in bold, square root of the AVE.

**Table 5:** Hypotheses testing results based on bootstrap path analysis (n = 5000).

Hypothesis	Path	β	Mean	Std. Dev	T Statistics	95% CI	*p* Values	Assumption
H1	PIIT → SE	0.16	0.166	0.168	0.954	[−0.296, 0.410]	0.34	No
H2	PIIT → PU	−0.065	−0.063	0.137	0.475	[−0.324, 0.227]	0.635	No
H3	PIIT → PEU	−0.008	0.007	0.165	0.049	[−0.338, 0.293]	0.961	No
H4	PIIT → BI	−0.125	−0.111	0.101	1.235	[−0.309, 0.084]	0.217	No
H5	SE → PU	0.293	0.295	0.106	2.763	[0.070, 0.490]	0.006	Yes
H6	SE → PEU	0.483	0.486	0.139	3.486	[0.145, 0.702]	0.000	Yes
H7	PCR → PU	0.002	0.011	0.096	0.024	[−0.180, 0.191]	0.981	No
H8	PCR → BI	0.163	0.176	0.115	1.418	[−0.081, 0.368]	0.156	No
H9	PEU → PU	0.248	0.246	0.125	1.99	[0.003, 0.488]	0.047	Yes
H10	PEU → PE	0.441	0.448	0.132	3.336	[0.125, 0.656]	0.001	Yes
H11	PEU → BI	0.211	0.211	0.118	1.782	[−0.037, 0.425]	0.075	No
H12	PU → BI	0.408	0.421	0.135	3.013	[0.137, 0.670]	0.003	Yes
H13	PE → BI	0.168	0.151	0.144	1.172	[−0.128, 0.425]	0.241	No
H14	PE → PU	0.419	0.42	0.143	2.924	[0.141, 0.701]	0.003	Yes

Note. Bootstrap results based on 5000 resamples. Paths are supported if the 95% confidence interval does not include zero.

**Table 6:** Effect size assessment.

Construct	BI	PE	PEU	PU	SE
PCR	0.045	-	-	0.000	-
PE	0.035	-	-	0.265	-
PEU	0.064	0.242	-	0.087	-
PIIT	0.032	-	0.000	0.008	0.026
PU	0.193	-	-	-	-
SE	-	-	0.296	0.139	-

Note. Values of 0.02, 0.15, and 0.35 depict small, medium, and large *f*^2^ effect sizes (Cohen, 1988).

**Table 7:** Significant indirect effects assessment.

Path	β	Mean	St Dev	T Statistic	*p* Value
SE → BI	0.342	0.354	0.083	4.124	<0.000
PEU → BI	0.251	0.25	0.089	2.821	0.005
PEU → PU	0.185	0.19	0.091	2.034	0.042
SE → PE	0.213	0.221	0.098	2.177	0.030
SE → PU	0.209	0.214	0.088	2.382	0.017
SE → PU → BI	0.119	0.124	0.061	1.965	0.049
SE → PEU → PE	0.213	0.221	0.098	2.177	0.030
PEU → PE →PU	0.185	0.19	0.091	2.034	0.042

Note. The upper section summarizes the total indirect effects, and the lower section summarizes the specific indirect effects.

## Data Availability

The data presented in this study are available on request from the corresponding author due to privacy restrictions.

## Appendix A

**Construct**	**Item**
Perceived Usefulness	PU1: Using AI tools enhance my teaching effectiveness.
	PU2: Using AI tools enhance the quality of my teaching.
	PU3: AI tools are useful in K-12 education.
Perceived Ease of Use	PEU1: Learning to use AI tools would be easy to me.
	PEU2: It is easy for me to become skillful at using AI tools.
	PEU3: My interaction with AI tools/platforms is clear and understandable.
Self-efficacy	SE1: I am confident that I can use AI tools effectively on many different tasks.
	SE2: When facing difficulties in using AI tools, I am certain that I will accomplish them.
Perceived Enjoyment	PE1: Using AI tools in teaching is enjoyable.
	PE2: Using AI tools in teaching is fun.
Perceived Cyber Risks	PCR1: The personal information that enters into AI tools/platforms will be handled securely.
	PCR2: AI tools/platforms have enough safeguards to make me feel comfortable using them to access educational services.
Personal Innovativeness in IT	PIIT1: If I heard about a new information technology, I would look for ways to experiment with it, especially AI.
	PIIT2: Among my peers, I am usually the first to try out new information technologies.
Intention to Use AI Tools	INT1: I intend to use AI tools for my studies in the future.
	INT2: I predict I would use AI tools for my teaching experiences.
	INT3: I plan to use AI tools frequently.
